# Single-cell RNA sequencing pseudobulk analysis and machine learning identify candidate biomarkers for ischemic cardiomyopathy

**DOI:** 10.3389/fcvm.2026.1840776

**Published:** 2026-07-29

**Authors:** Xianhua Ye, Guoxiang Wu, Jialan Xie, Yanqing Wu, Daqiu Chen, Yixing Chen, Shanghua Xu, Shunxiang Luo

**Affiliations:** 1 Department of Cardiology, Nanping First Hospital affiliated to Fujian Medical University, Nanping, Fujian, China; 2 Department of Gastroenterology, Nanping First Hospital affiliated to Fujian Medical University, Nanping, Fujian, China

**Keywords:** biomarkers, feature selection, ischemic cardiomyopathy, machine learning, pseudobulk, scRNA-seq

## Abstract

**Background:**

Ischemic cardiomyopathy (ICM) is a condition characterized by inadequate blood supply to the coronary arteries, resulting in myocardial damage and decreased cardiac functionality. This study aimed to identify potential biomarkers and regulatory networks in ICM, providing a foundation for further mechanistic and therapeutic investigations.

**Methods:**

We analyzed public single-cell RNA sequencing (scRNA-seq) data to identify cell subpopulations through dimensionality reduction clustering followed by manual annotation. Differentially expressed genes (DEGs) were derived using the pseudobulk method. Subsequently, we employed three machine learning algorithms combined with the Boruta feature selection approach to screen for disease-characteristic genes in an external ICM dataset. Potential regulatory networks were reconstructed by predicting transcription factors (TFs) and microRNAs (miRNAs). Finally, we validated the expression levels of signature genes, TFs, and miRNAs in an *in vivo* ICM rat model.

**Results:**

The pseudobulk analysis identified 168 DEGs, with machine learning selecting four hub genes as key signatures demonstrating acceptable ICM discriminative power. Their area under the curve (AUC) values were as follows: MLLT3 (76.7%), GFOD1 (78.6%), COLEC12 (77.8%), and RARRES1 (79.9%). Notably, when combined into a four-gene signature, a substantially higher AUC of 93.0% was achieved for the discrimination of ICM. Transcription factor analysis delineated that GFOD1, MLLT3, RARRES1, and COLEC12 were regulated by 17, 7, 7, and 3 TFs, respectively. The CTCF was found to be a shared transcription factor. Computational miRNA analysis retrieved 329 miRNAs. *In vivo* validation studies confirmed significantly reduced expression of four signature genes and CTCF, along with elevated levels of miR-195-5p and miR-5680 in ICM samples compared to those in sham controls.

**Conclusion:**

Our study characterized GFOD1, MLLT3, COLEC12, RARRES1, miR-195-5p, and miR-5680 as promising biomarkers for ischemic cardiomyopathy, with CTCF acting as a candidate transcription factor.

## Introduction

Approximately 64 million patients are estimated to have heart failure worldwide, with the prevalence of heart failure in China being around 1.3% (1). Ischemic cardiomyopathy is the most common cause of heart failure (2). Its pathogenesis involves myocardial ischemia-induced cellular injury and death, oxidative stress, inflammatory cascades, apoptosis, necrosis, ventricular remodeling, and metabolic dysregulation (3–5). These mechanisms collectively contribute to progressive impairment of cardiac structure and function, culminating in heart failure.

Although progress has been made in understanding the mechanisms of heart failure, specific cell types and regulatory molecules involved in ischemic heart disease are still under investigation (6). Despite advances in revascularization and pharmacotherapy, ICM remains associated with high morbidity and mortality, underscoring the urgent need for novel diagnostic and therapeutic strategies (7).

Recent advances in scRNA-seq have revolutionized our understanding of cardiac cellular diversity and disease-specific transcriptional alterations (8). For example, scRNA-seq studies have showed dysregulated endothelial cell signaling and fibroblast activation as key contributors to myocardial fibrosis in the ICM (9, 10). However, most prior bulk transcriptomic analyses overlook cell-type-specific gene expression patterns, potentially masking critical biomarkers (11). Concurrently, machine learning algorithms, including least absolute shrinkage and selection operator (LASSO) and random forest (RF), have emerged as powerful tools for high-dimensional data mining, facilitating the extraction of diagnostic signatures from complex omics datasets (12, 13). Despite these technological strides, to our knowledge, few studies have integrated scRNA-seq pseudobulk analysis with machine learning-guided feature selection to dissect ICM-specific molecular networks, leaving a gap in precision biomarker discovery (14). Furthermore, regulatory networks involving transcription factors and miRNAs, which are key modulators of post-ischemic gene expression, are poorly characterized in the ICM (15, 16).

**Figure 1:**
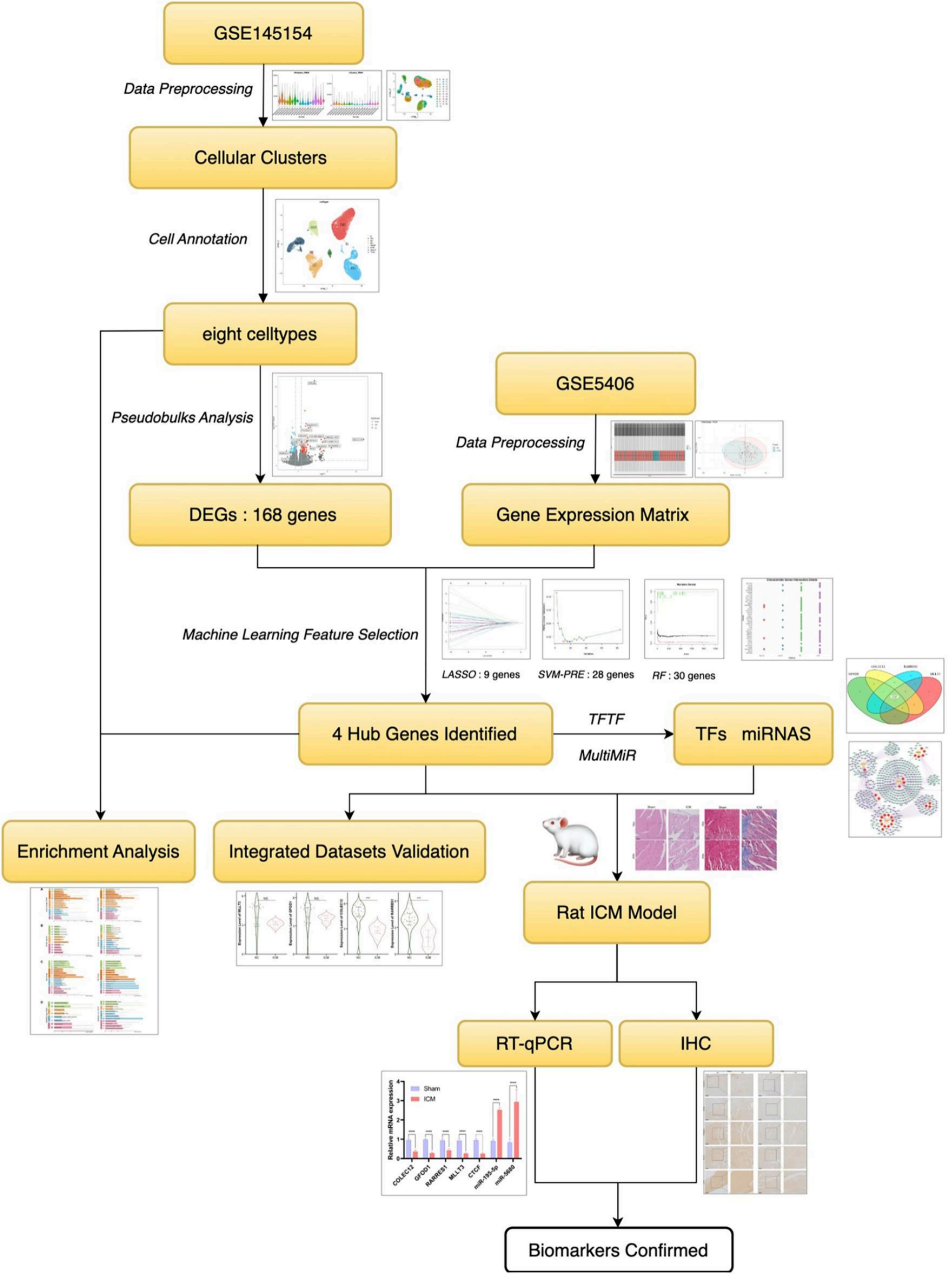
Research workflow. Integrated bioinformatics analysis and experimental validation.

In this study, we bridge these knowledge gaps by combining scRNA-seq pseudobulk analysis with machine learning-driven feature selection to uncover specific biomarkers for ICM. Utilizing a public scRNA-seq dataset (GSE145154) and a microarray RNA-seq dataset (GSE5406), we first delineated transcriptional changes across cardiac cell subpopulations in ICM. Pseudobulk differential analysis and cross-dataset validation were implemented to pinpoint differentially expressed genes (17). Next, the LASSO, support vector machine with recursive feature elimination (SVM-RFE), RF, and Boruta method were integrated to prioritize hub genes with diagnostic potential. We further constructed TF-miRNA-mRNA regulatory networks to uncover upstream modulators of ICM progression. Finally, *in vivo* validation in a rat coronary ligation ICM model confirmed the dysregulation of these candidate biomarkers at both the mRNA and protein levels. The overall research workflow was presented in Figure 1, which illustrated the multi-stage analytical pipeline from single-cell RNA sequencing data processing to experimental validation in a rat ICM model. Our integrated bioinformatics analysis and experimental validation not only detected ICM-associated genes (GFOD1, MLLT3, COLEC12, RARRES1) and miRNAs (miR-195-5p, miR-5680) but also implicated CTCF as a central transcriptional regulator, offering new insights into ICM pathogenesis and therapeutic targeting.

## Methods

All methods were performed in accordance with the relevant guidelines and regulations. All methods for animal experiments are reported in accordance with ARRIVE guidelines (https://arriveguidelines.org).

### Data acquisition and preprocessing

Both the scRNA-seq dataset (GSE145154) and the microarray dataset (GSE5406) include left ventricular myocardial samples from non-heart failure controls (NC) and ICM patients. These datasets were downloaded from the Gene Expression Omnibus (GEO) database (http://ncbi.nlm.nih.gov/geo) (18). The GSE145154 dataset includes 12 ICM patient samples and 2 non-heart failure control samples, and the GSE5406 dataset contains 108 ICM patient samples and 16 normal control samples. Both datasets are derived from left ventricular myocardial tissue. The details of the datasets are listed in Supplementary Table 1.

The scRNA-seq data processing and analysis were conducted using Seurat (v5.1.0) within the R environment (v4.3.3). The workflow included quality control metrics, exploratory data analysis, and statistical evaluation. Initially, low-quality cells were filtered out using the specific criteria: (1) genes detected in fewer than 5 cells; (2) cells with fewer than 300 detected genes; (3) cells with mitochondrial gene expression of 25% or higher; and (4) cells with ribosomal gene expression of 3% or lower. Following data normalization, we employed the FindVariableFeatures function to pinpoint the most variable genes between cells. Principal component analysis (PCA) was then conducted to identify noticeable principal components for linear dimensionality reduction with a significance threshold of *P* < 0.05. RunHarmony was applied to integrate multiple single-cell samples while mitigating batch effects, ensuring data consistency across samples. Cellular clusters were delineated using the FindNeighbors and FindClusters functions at a resolution of 0.8, followed by uniform manifold approximation and projection (UMAP) for visualization. Cell types within the left ventricle were classified and annotated manually according to biological context and the expression patterns of marker genes (refer to Supplementary Table 2 for cell surface marker genes).

For the microarray data analysis, quantile normalization was performed through the limma package to obtain a standardized gene expression matrix. Quality control was conducted by clustering tree analysis and PCA, with outlier samples removed if detected.

### Pseudobulk differential analysis of scRNA-seq data

The prevailing method for single-cell differential analysis utilized the FindMarkers function, primarily employing the Wilcoxon rank-sum test. However, this approach tends to prioritize sample-specific highly expressed genes, which strictly reflect individual differences. Pseudobulk was a method employed in single-cell transcriptomics to aggregate data from multiple single-cell samples into two or more groups, thereby allowing for conventional transcriptomic differential expression analysis. This approach involved summing the expression values across cells within each group, creating a pseudobulk expression matrix that can be analyzed by standard bulk RNA-seq differential gene expression tools such as DESeq2.

DESeq2 was performed using a negative binomial generalized linear model with the Wald test, followed by Benjamini-Hochberg multiple testing correction, with false discovery rate (FDR)-adjusted *P* < 0.05, enabling the identification of more reliable differentially expressed genes. To identify genes or signatures capable of distinguishing ischemic cardiomyopathy at the tissue level, whole-cell aggregation in pseudobulk was performed after the major cell subpopulations had been classified in the present study.

### Machine learning and feature genes selection

In the standardized expression matrix of the ICM gene chip dataset GSE5406, feature variable selection was performed via three machine learning algorithms (including LASSO, SVM-RFE and RF) and the Boruta method. The LASSO was a regression analysis method that incorporated an L1 regularization term into the model, thereby compressing the weights of unimportant features to zero and achieving feature selection. The SVM-RFE was a recursive feature elimination method derived from a support vector machine (19). This method recursively removed the least important features, progressively optimizing the model to ultimately select an optimal set of features. The RF evaluated the contribution of each feature to the model's decision-making process to assess its impact. The Boruta was a robust wrapper method anchored in random forest classification, that systematically evaluated feature importance by comparing original attributes with permuted shadow features through iterative importance scoring. The overlapping genes detected by all algorithms were designated as the signature genes for ICM. ROC curve analysis was performed to evaluate the predictive ability of candidate genes in discriminating ICM patients from controls in the GSE5406 dataset. We calculated the area under the curve, and an AUC > 0.7 was considered acceptable.

### Enrichment analysis in gene expression-defined cell subgroups

Single-cell expression patterns of signature genes were visualized across distinct cell types in the GSE145154 dataset. The cellular populations were stratified into high-expression and low-expression subgroups according to signature genes expression levels, with the median value used as the cutoff. Subsequently, enrichment analysis was conducted for Gene Ontology [GO; including biological processes [BP], cellular components [CC], and molecular functions [MF]], Kyoto Encyclopedia of Genes and Genomes (KEGG), and Disease Ontology (DO). This analytical framework enabled systematic evaluation of how target gene expression dynamics influence critical biological pathways and molecular functions. Enrichment analysis was performed by employing the clusterProfiler package (version 4.10.1) (20) with a significance threshold of *P* < 0.05, with multiple testing correction applied by the Benjamini-Hochberg method to control the false discovery rate.

### Expression validation in integrated single-cell datasets

The GSE145154 dataset was integrated with additional normal human cardiac scRNA-seq datasets (GSE134355, GSE183852, GSE185100, and GSE159929). The detailed information on the datasets was provided in Supplementary Table 1. After quality control and harmony-enabled batch correction, a pseudobulk matrix was generated through row sum aggregation and CPM normalization. We compared the expression levels of signature genes between the ICM and NC groups.

### TF-mRNA-miRNA regulatory network

Transcription factors and miRNAs were crucial regulators of mRNA expression, modulating target genes through transcriptional and post-transcriptional mechanisms, respectively. For TF-target prediction, we utilized the Transcription Factor Target Finder (TFTF) R package (21), which integrates binding evidence from four authoritative databases: hTFtarget, JASPAR, GTRD, and ChIP_Atlas in this study. This multi-database approach ensured comprehensive coverage of potential TF-mRNA interactions, with all predictions being contextualized by incorporating heart tissue-specific expression data from GTEx. For mRNA-miRNA interactions, we implemented the multiMiR (22) R package (v1.24.0), a robust tool that aggregates predictions from multiple established databases, including both computationally predicted and experimentally validated resources. In this study, the databases utilized included DIANA-microT, ElMMo, MicroCosm, miRanda, miRDB, miRTarBase, PITA, TarBase, and TargetScan. This integrative strategy enhances prediction reliability by combining multiple computational and experimentally validated interactions. The resulting TF-mRNA and mRNA-miRNA interactions were imported into Cytoscape software (version 3.10), an open-source platform for network visualization and analysis, to construct and visualize the comprehensive TF-mRNA-miRNA regulatory network.

### Ischemic cardiomyopathy rat model

Eight male rats (8–10 weeks old, weighing 240–260 g, specific pathogen-free) were obtained from the Shanghai SIPPR BK Laboratory Animals Ltd. They were housed in an environment maintained at a temperature of 20-22 °C, humidity of 55%, and a 12-hour light/dark cycle, with free access to food and water. The random number table was used to randomly divide the rats into a sham surgery control group and an ICM group (*n* = 4/group). The ICM model in rats was established by permanently ligating the left anterior descending branch of the coronary artery. Sham-operated rats were subjected to a similar surgical procedure without ligation of the left anterior descending branch of the coronary artery. No animals died after surgery. An echocardiography examination was conducted using a Vevo 2100 system (Visual Sonics) after 1 week. Initially, a B-mode long-axis view was captured by aligning the probe parallel to the long axis of the left ventricle. The probe was subsequently rotated by 90° to acquire an M-mode short-axis view of the left ventricle. Echocardiographic recordings encompassed a minimum of two complete cardiac cycles. The echocardiographic parameters, including left ventricular fractional shortening (LVFS), left ventricular ejection fraction (LVEF), left ventricular internal diameter at end-diastole (LVIDd), and left ventricular internal diameter at end-systole (LVIDs), were analyzed utilizing Vevo2100 cardiac analysis software. The mean values were determined founded on measurements taken over three consecutive heartbeats. The serum levels of B-type natriuretic peptide (BNP) were measured in both the NC and ICM groups of rats using a rat brain natriuretic peptide enzyme-linked immunosorbent assay (ELISA) kit. Four weeks after LAD ligation, all rats were euthanized under deep anesthesia. The hearts were rapidly excised, and left ventricular tissues containing the infarct region were collected for further analysis. Myocardial pathological changes were detected through conventional hematoxylin-eosin (HE) staining kits and Masson's trichrome staining kits.

### Real time quantitative polymerase chain reaction

We determined the mRNA expression levels of the hub genes using RT-qPCR. Total RNA was extracted from ventricular tissues with TRIzon Reagent (CW0580) (CWBIO, China). This RNA was then reverse transcribed into complementary DNA with a reverse transcription HiFiScript kit from CWBIO. RT-qPCR was performed in 20 μL reactions via SYBR Green Master Mix with gene-specific primers under the following cycling conditions: 95 °C for 10 min, followed by 40 cycles of 95 °C for 15 s and 60 °C for 30 s. Data were analyzed automatically by the real-time PCR system. The primers used for RT-qPCR were listed in Table 1. The relative mRNA expression of specific genes or miRNAs was quantified by the 2^−△△CT^ method and normalized to *β*-actin for mRNA or U6 for miRNA.

**Table 1:** The primers for RT-qPCR analysis of mRNA and miRNA targets.

Primers-F	5'→3'	Primers-R	5'→3'
GFOD1	CACCTACATCATCGATCTGCTT	GFOD1	CTTGACAAAGGTCTTGAGCAAC
COLEC12	ACTGTGCTGGATTGATTTATGC	COLEC12	GAGGCATCAGAAAATACGTTCC
MLLT3	GCAGAGACCATACTTGACAGTA	MLLT3	ATGGCGATGCTCTAAATAGTCA
RARRES1	CAGTTTCTGCACTACTACCTGA	RARRES1	TACCAGACCATATGAATACGGC
CTCF	GTGATGCTGTGTTTCATGAGAG	CTCF	CAAAGTTGGGATCATGATAGCG
miR-195-5p	GCGCGTAGCAGCACAGAAAT	miR-195-5p	AGTGCAGGGTCCGAGGTATT
miR-5680	CGCGGAGAAATGCTGGACTA	miR-5680	AGTGCAGGGTCCGAGGTATT

### Immunohistochemistry

Ventricular tissues were fixed in formalin, paraffin-embedded, and sectioned at 5 μm. After deparaffinization and antigen retrieval in EDTA buffer (microwave heating, 20 min), endogenous peroxidase activity was blocked with 3% H₂O₂ at room temperature (10 min). Sections were incubated overnight at 4 °C with primary antibodies against GFOD1 (Atlas), COLEC12 (Affinity), MLLT3, RARRES1 and CTCF (Proteintech), diluted according to the manufacturer's instructions, followed by secondary antibody incubation (30 min) and DAB visualization. Counterstaining with hematoxylin, dehydration, and mounting were performed as previously described. Images were acquired using an Olympus microscope with optimal fields selected for analysis. Protein expression was assessed by measuring the percentage of positive cells in selected high-power fields (×200 magnification) in Fiji software.

### Statistical analysis

All the statistical analyses were performed in R software (version 4.3.3) and GraphPad Prism software (version 9.1.0). Normality was assessed through the Anderson–Darling test, whereas homogeneity of variance was evaluated through Levene's test. For normally distributed data, the t-test was used for intergroup comparisons. For data that were not normally distributed, the Wilcoxon rank-sum test was applied. A threshold of *P* < 0.05 was considered statistically significant.

## Results

### Single-cell transcriptome landscape and cell annotation

**Figure 2:**
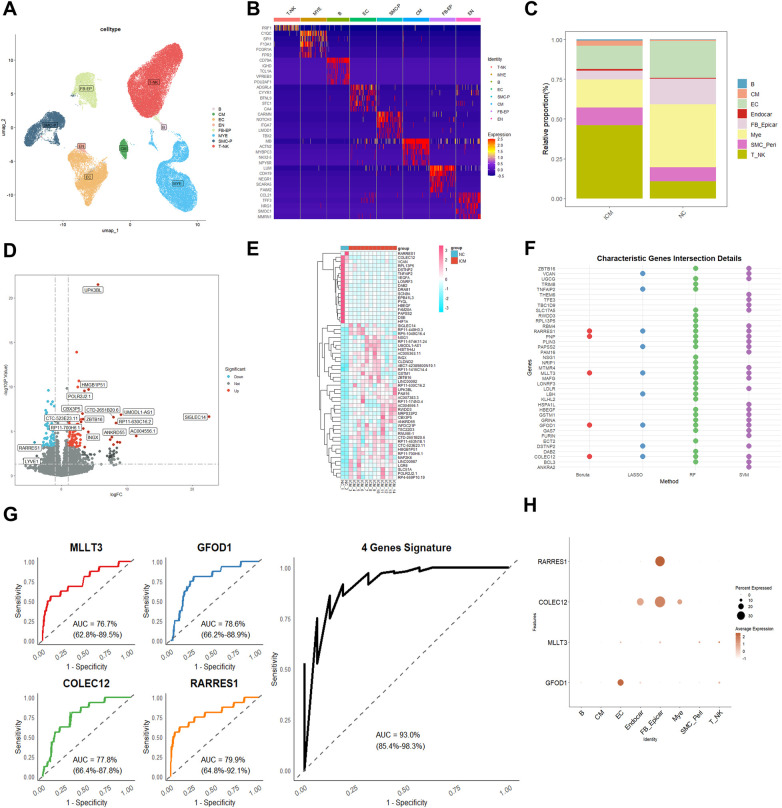
Single-cell transcriptomic profiling, pseudobulk DEG analysis, and machine learning-driven ICM signature identification. **(A)** UMAP projection of all left ventricular tissue cells from the GSE145154 dataset, colored by manually annotated cell types. **(B)** Heatmap of the top 5 cell-type-specific marker genes in eight cardiac cell populations. Gene expression levels was color-coded (dark blue: lowest; red: highest). **(C)** Comparative analysis of cell-type proportions between ICM and nonfailing controls. **(D)** Pseudobulk analysis of the GSE145154 dataset showed differential gene expression in ICM vs. nonfailing controls (DESeq2, |log₂FC|> 1, FDR < 0.05). **(E)** Heatmap visualization of stringently selected DEGs (|logFC|> mean + 2SD) illustrated distinct intergroup expression patterns. **(F)** Machine learning-driven identification of ICM signature genes in the GSE5406 dataset. **(G)** ROC analysis and diagnostic validation of candidate genes and the four-gene combined model for ICM inferred from the GSE5406 dataset. **(H)** Bubble plot displays the expression heterogeneity of signature genes across different cell types in the GSE145154 dataset.

We downloaded the GSE145154 scRNA-seq dataset from the GEO database. After quality control, 60,996 left ventricular myocardial cells remained suitable for further analysis. Eight cell types were annotated manually, including cardiomyocytes (CM), endothelial cells (EC), endocardial cells (EN), fibroblast-epicardial cells (FB-EP), myeloid immune cells (MYE), smooth muscle cells-pericytes (SMC-P), T-NK cells (T-NK), and B cells (B) (Figure 2A). The top 5 marker genes for each cell cluster were displayed in the heatmap (Figure 2B). The ICM group presented increased proportions of cardiomyocytes and T-NK cells, whereas the proportions of myeloid immune cells, fibroblasts-epicardial cells, and endocardial cells decreased (Figure 2C).

### Determination of DEGs through pseudobulk

We employed the pseudobulk method to stratify single-cell data into the NC and ICM groups. Differential expression analysis using DESeq2 revealed 168 significant DEGs (adjusted *P* < 0.05, |log2FC|>1) between the ICM and NC groups. The DEGs were visualized in a volcano plot (Figure 2D), highlighting 99 upregulated and 69 downregulated genes. DEGs with |logFC| values exceeding the cutoff (mean + 2SD of |logFC| values) were selected for heatmap visualization. The heatmap displayed their expression patterns and intragroup consistency (Figure 2E).

### Machine learning-powered identification of signature genes in ICM

We applied LASSO, SVM-RFE, and RF machine learning algorithms combined with the Boruta method to screen 168 candidate genes in the GSE5406 dataset, which includes left ventricular myocardial samples from 108 ICM patients and 16 normal individuals. Through consensus feature selection (see Supplementary Material 2), we yielded four hub genes: GFOD1, MLLT3, COLEC12 and RARRES1 (Figure 2F). ROC curve analysis demonstrated acceptable predictive performance of the four hub genes for ICM, with AUC values of 76.7% (MLLT3), 78.6% (GFOD1), 77.8% (COLEC12), and 79.9% (RARRES1) (Figure 2G). A four-gene signature achieved an AUC of 93.0% for distinguishing ICM, markedly higher than the AUC of any single gene (Figure 2G). Subsequently, bubble plots were generated to visualize the expression patterns of these hub genes across different cell types in the GSE145154 dataset (Figure 2H). MLLT3 was ubiquitously expressed across all cell types, whereas GFOD1 was enriched predominantly in endothelial cells. Both COLEC12 and RARRES1 were specifically expressed in fibroblast-epicardial cells, with COLEC12 additionally detected in myeloid immune cells and endocardial cells.

### Functional characterization of cell subgroups stratified by target gene expression

**Figure 3:**
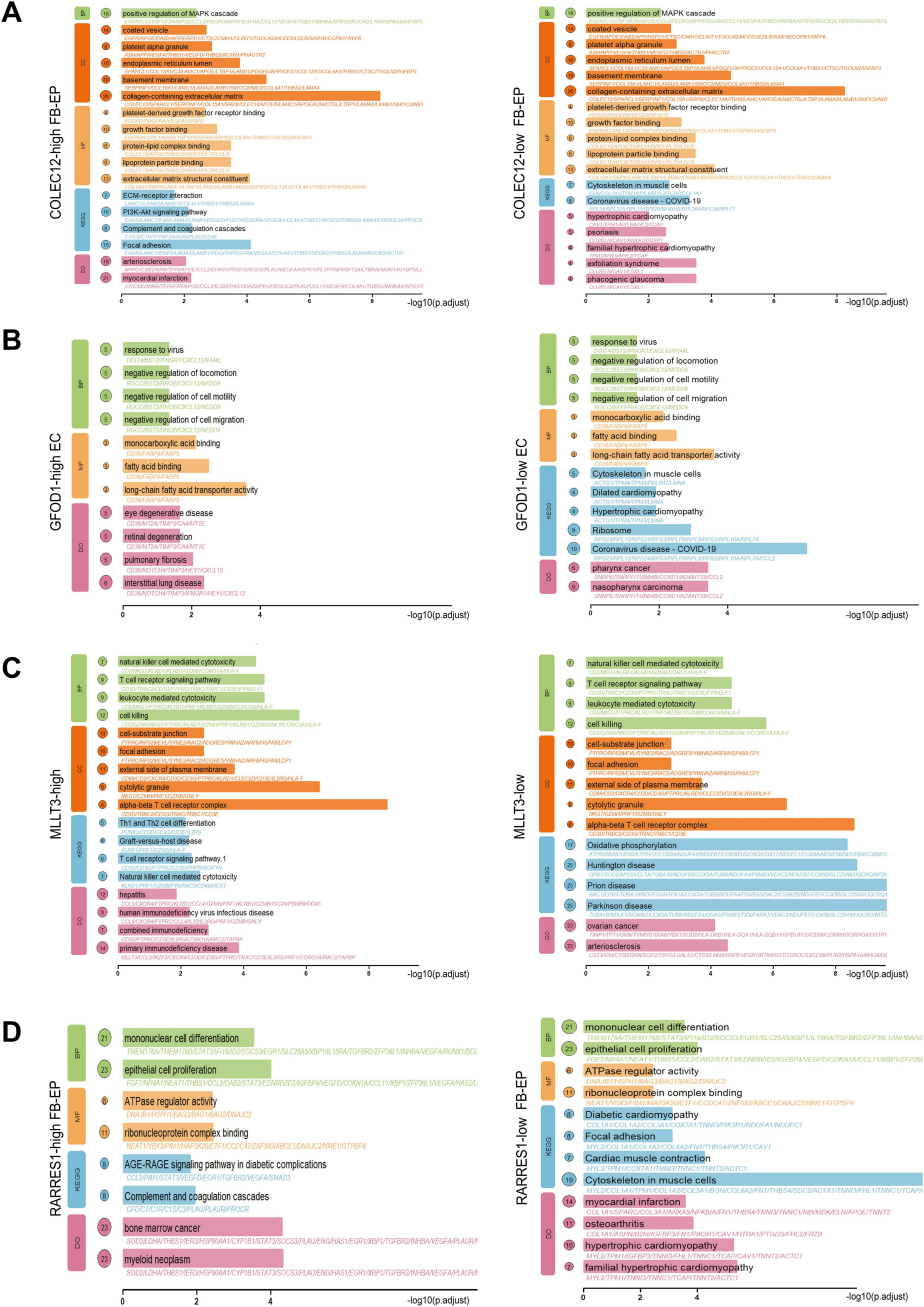
Functional enrichment analysis (GO, KEGG, and DO) of target gene-high and target gene-low cell subpopulations. lime green = BP, vivid orange = CC, sunset orange = MF, sky blue = KEGG, blush pink = DO. **(A)** COLEC12-high FB-EP and COLEC12-low FB-EP cells. **(B)** GFOD1-high EC and GFOD1-low EC cells. **(C)** MLLT3-high and MLLT3-low cells. **(D)** RARRES1-high FB-EP and RARRES1-low FB-EP cells.

Compared with cells with high expression of target genes, enrichment analysis showed that GFOD1-low ECs were strongly enriched in coronavirus disease, cytoskeleton in muscle cells, hypertrophic cardiomyopathy, and cardiomyopathy pathways (KEGG), and nasopharyngeal carcinoma (DO) (Figure 3B). Similarly, COLEC12-low FB-EP and RARRES1-low FB-EP were also associated with the cytoskeleton in muscle cells and hypertrophic cardiomyopathy (Figures 3A, D). Additionally, RARRES1-low FB-EP was markedly linked to the AGE-RAGE signaling pathway in diabetic complications, complement and coagulation cascades (KEGG), and myocardial infarction (DO) (Figure 3D). MLLT3-low cells were distinctly correlated with oxidative phosphorylation (KEGG) and arteriosclerosis (DO) (Figure 3C).

### Expression levels of the hub genes in the integrated dataset

**Figure 4:**
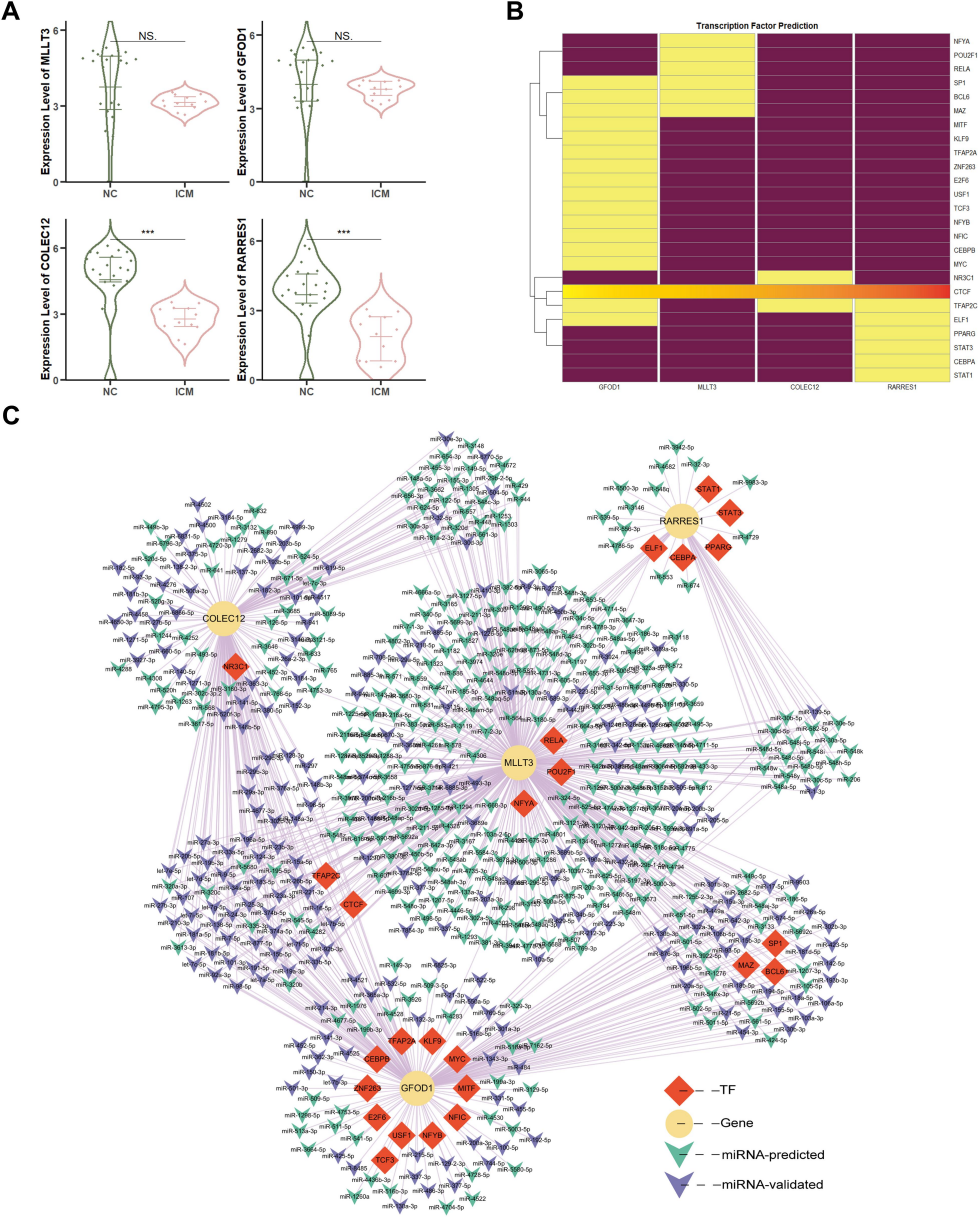
Key genes expression and regulatory network. **(A)** Violin plots showing expression differences of key genes between ICM patients and nonfailing controls. Statistical significance was assessed using the two-sided Wilcoxon rank-sum test. **(B)** Predicted transcription factors of key genes utilizing TFTF analysis. **(C)** The miRNA-mRNA-TF regulatory network visualization in Cytoscape. red = TF, yellow = Gene, green = miRNA-perdicted, green = miRNA-validated. *NS > 0.05, *P < 0.05, **P < 0.01, ***P < 0.001.

Integrated single-cell dataset analysis pinpointed that the gene expression levels of COLEC12 and RARRES1 were significantly lower in the ICM group than in the NC group. Although no statistically significant differences were observed in the mRNA expression levels of MLLT3 and GFOD1 between the two groups, a consistent downward trend was noted in the ICM group (Figure 4A).

### Analyzing TF and miRNA targets via TFTF and multiMiR

Our TFTF-assisted analysis manifested differential transcriptional regulation of target genes, with distinct patterns of transcription factor binding observed for GFOD1 (regulated by 17 TFs), MLLT3 (7 TFs), RARRES1 (7 TFs), and COLEC12 (3 TFs). Intriguingly, CTCF emerged as a shared transcription factor for these genes (Figure 4B). Through the multiMiR package, a total of 551 unique miRNAs that may regulate the expression of the four signature genes were identified, including 329 predicted miRNAs and 222 validated miRNAs. These corresponded to 770 unique miRNA–mRNA interaction pairs, which comprised 367 predicted and 403 validated unique miRNA–mRNA interactions. Our integrated analysis uncovered a complex TF-mRNA-miRNA regulatory network (Figure 4C). The network of TF-mRNA-miRNA interactions was detailed in Supplementary Table 3. We did not initially validate all 329 predicted miRNAs in ICM. Given that RARRES1 exhibited the highest AUC among the four hub genes, we prioritized the RARRES1-targeting miRNAs for further validation. Of the 329 miRNAs, 39 were found to target RARRES1, including 32 predicted and 7 validated miRNAs. A literature search in PubMed was performed for the 32 predicted miRNAs. Based on the literature results, miR-5680 and miR-195-5p were manually selected as candidate regulators of RARRES1 for experimental validation (see Supplementary Material 3 for detailed miRNAs screening results).

### Establishment of ischemic cardiomyopathy rat model

**Figure 5:**
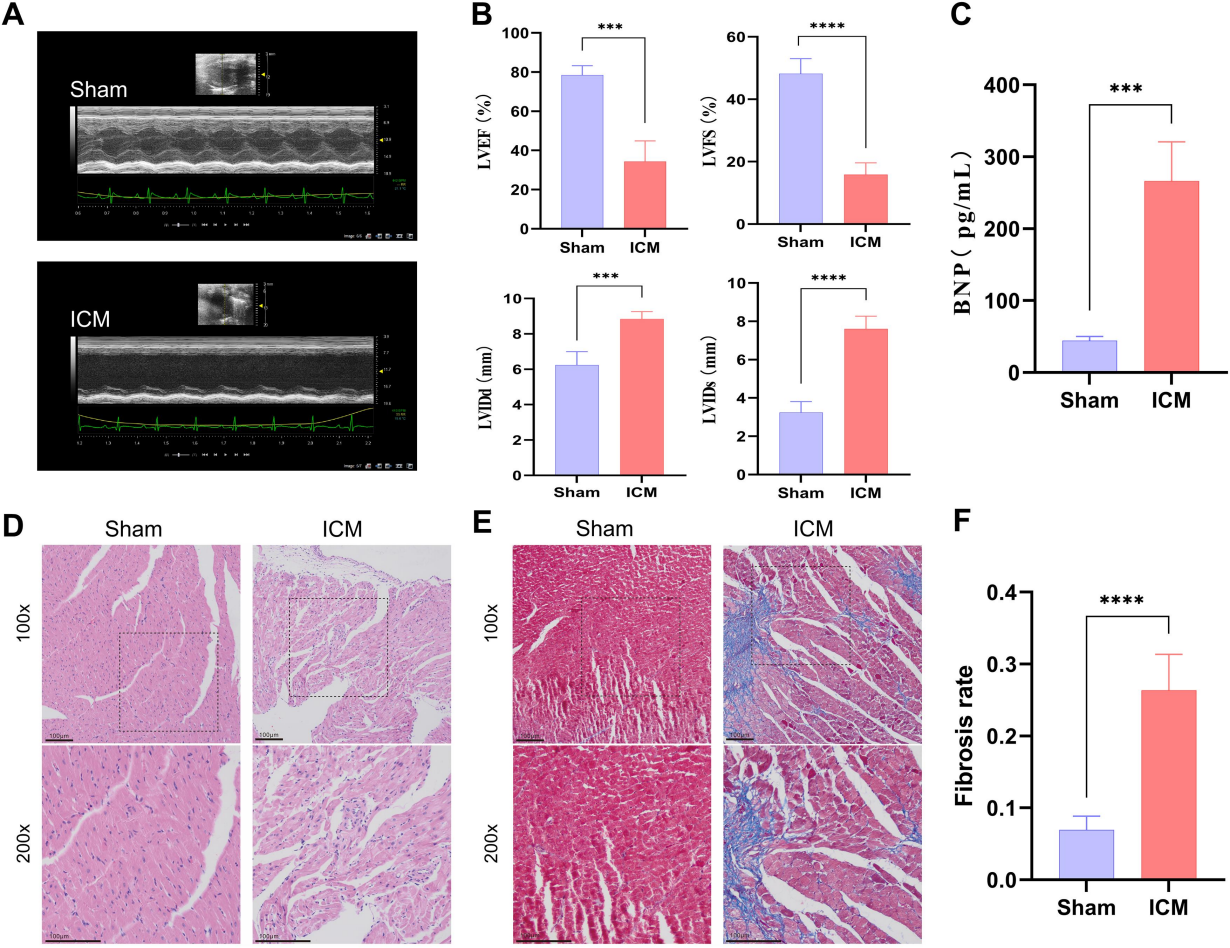
Establishment of the experimental rat ICM model. **(A)** Representative M-mode echocardiographic tracings in each group (*n* = 4). **(B)** Quantitative analysis of echocardiographic parameters in each group (*n* = 4). **(C)** Serum BNP concentrations measured by ELISA (*n* = 4). **(D)** Representative HE-stained sections. **(E)** Representative Masson-stained sections. **(F)** Fibrosis rates in each group. A total of 6 microscopic fields were analyzed per group (2 biological replicates×3 technical replicates per biological replicate). Statistical comparisons between two groups were performed using two-tailed unpaired Student's t-test. *P < 0.05, **P < 0.01, ***P < 0.001, ****P < 0.0001 vs. the sham group.

In ischemic cardiomyopathy rat model, the ICM group exhibited poor cardiac morphology and function (Figure 5A). According to echocardiographic evaluation, the ICM group began to show considerable reductions in LVEF and LVFS compared with the sham group, whereas the LVIDs and LVIDd were markedly increased in the ICM group (Figure 5B). The serum BNP levels in the ICM group were significantly elevated (Figure 5C). H&E staining illustrated well-preserved myocardial architecture with tightly aligned cardiomyocytes in the sham group, whereas the ICM group displayed disrupted tissue integrity characterized by a loose extracellular matrix and prominent inflammatory infiltration (Figure 5D). Masson's trichrome staining quantitatively confirmed markedly increased collagen deposition and severe interstitial fibrosis in ICM hearts compared to those of sham controls (Figures 5E, F). In light of these results, we successfully established an ICM model in rats.

### Assessment and validation of biomarker values

**Figure 6:**
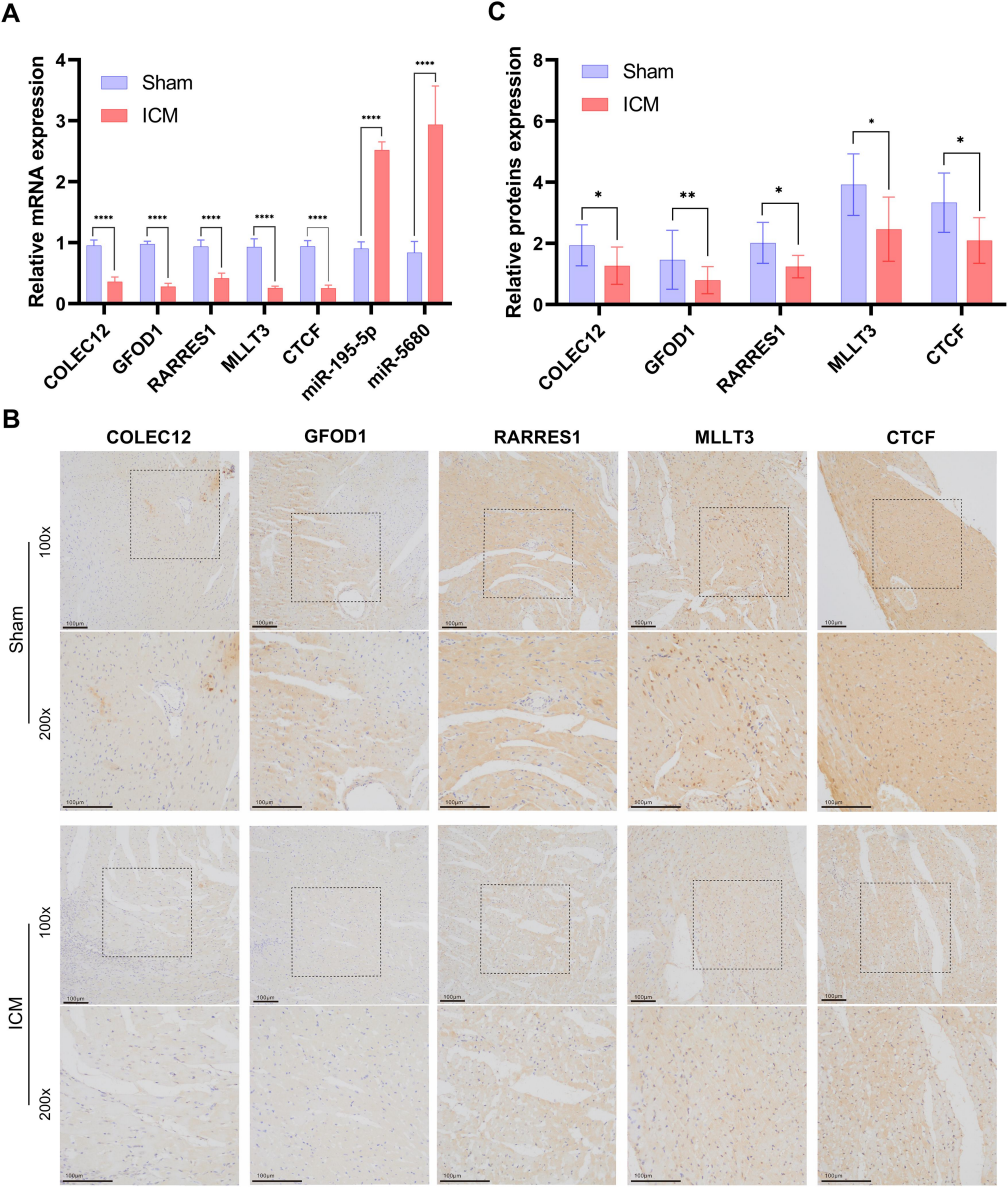
Biomarkers validation in the rat ICM model. **(A)** Relative mRNA expression levels of the 7 biomarkers. **(B)** Representative IHC sections. **(C)** Relative protein expression levels of the 5 biomarkers in each group. For both mRNA and protein analyses, data were obtained from 6 replicates per group (2 biological replicates×3 technical replicates per biological replicate). Statistical comparisons between two groups were performed using two-tailed unpaired Student's t-test. *P < 0.05, **P < 0.01, ***P < 0.001, ****P < 0.0001 vs. the sham group.

The four characteristic genes, the transcription factor CTCF, miR-195-5p, and miR-5680 were further validated in the rat ICM model. RT-qPCR analysis evidenced significant upregulation of miR-195-5p and miR-5680, accompanied by marked downregulation of GFOD1, MLLT3, COLEC12, RARRES1 and CTCF mRNA expression in ICM samples compared with controls (Figure 6A). Immunohistochemical analysis consistently elucidated significantly reduced protein expression levels of these targets (GFOD1, MLLT3, COLEC12, and RARRES1) along with the transcription factor CTCF (Figure 6B), as quantified by average optical density measurements (Figure 6C). These experimental findings validated our prior bioinformatics predictions in the integrated single-cell dataset analysis.

## Discussion

Heart failure is a global disease that imposes a significant health burden worldwide. Ischemic cardiomyopathy is a leading cause of heart failure (23). Current treatments for ICM have limitations, highlighting the need for novel therapeutic strategies (24). Our study leverages single-cell RNA sequencing data and machine learning to uncover potential biomarkers and regulatory networks in ICM, contributing to the existing body of knowledge.

The public dataset GSE5406 includes 108 ICM samples and 16 normal controls, representing an ICM dataset with a relatively large sample size currently available. In a preliminary analysis, we attempted to split the GSE5406 dataset into a training set and an independent test set at a 7:3 ratio. In the training set (76 ICM, 12 normal), we performed DEG analysis followed by machine learning-based feature selection, which yielded similar characteristic gene results. However, when we further validated the final four genes using ROC curve analysis in the test set (32 ICM, 4 normal), we obtained larger AUC values (see Supplementary Material 4), compared to the ROC analysis in the full dataset. Considering the potential risk of overfitting, we ultimately chose the more conservative result based on the full dataset.

To the best of our knowledge, the methodological novelty of combining scRNA-seq pseudobulk analysis with machine learning (LASSO/SVM-RFE/RF + Boruta) has not been previously applied to discover ICM biomarkers. This strategy reduces false discoveries arising from cell sparsity and better reflects true biological variation across samples. Our bioinformatics analysis pinpointed four signature genes with acceptable discriminative potential for ICM (AUC >0.7). Glucose-fructose oxidoreductase domain-containing 1 (GFOD1) has been classified within the glucose-fructose oxidoreductase, inositol dehydrogenase, and rhizopine catabolism protein MOCA (Gfo/Idh/MocA) family (25), although crystallographic studies revealed its homodimeric structure lacks critical NAD/NADP cofactor-binding residues, suggesting pseudoenzyme characteristics (26). Prior reports linked this gene to attention-deficit/hyperactivity disorder (ADHD) (27), and renal clear cell carcinoma progression (28). Its potential as a gene biomarker for the diagnosis of acute myocardial infarction was also noted (29). Furthermore, previous studies reported that GFOD1 was implicated in the metabolic pathways of acute myocardial infarction, a condition characterized by continuous transcriptome-level biological changes (29). However, those findings came from peripheral blood samples (GSE66360, GSE48060, GSE29532), whereas we analyzed myocardial tissue. Collectin subfamily member 12 (COLEC12 or CL-12), a pattern recognition molecule (PRM) of the innate immune system encoded by chromosome 18p11.32 (30), belonged to the collectin protein family and primarily mediated immune responses and cellular recognition (31). Previous studies established its roles in pathogen recognition (32) and macrophage activation (33), with additional associations reported in gastric cancer (34, 35) and Alzheimer's disease (36). Our findings aligned with recent work linking COLEC12 to vascular inflammation and atherosclerosis progression (37), as supported by our DO enrichment analysis. This observation was mechanistically plausible given that RBPJ-mediated chromatin remodeling governs macrophage efferocytosis capacity, where disruption of this program impairs phagocytic gene expression and promotes defective tissue clearance (38). Defective efferocytosis has been consistently associated with adverse post-ischemic cardiac remodeling, including increased fibrosis and impaired ventricular function (39, 40). Thus, altered COLEC12 expression in ICM myeloid cells may serve as a marker for epigenetically dysregulated states that were prone to impaired efferocytosis and adverse post-ischemic remodeling (41).

The myeloid/lymphoid or mixed-lineage leukemia translocated to 3 (MLLT3), a key YEATS family chromatin-binding protein, functioned as an essential component of the super elongation complex (42). As a critical regulator of hematopoiesis, MLLT3 formed the leukemogenic MLL-MLLT3 fusion gene upon translocation, directly driving acute leukemogenesis (43). Moreover, MLLT3 expression levels were correlated with invasiveness, proliferation, and poor prognosis in melanoma (44) and lung adenocarcinoma (45). The reduced myeloid immune cell proportions observed in ICM may reflect macrophage loss driven by mitochondrial dysfunction, as recent work has shown that SerpinB2-regulated oxidative phosphorylation is essential for tissue-resident macrophage survival under chronic inflammatory stress (46). This aligns with our enrichment analysis linking MLLT3-low cells to oxidative phosphorylation and arteriosclerosis pathways, suggesting that macrophage metabolic impairment could contribute to reduced macrophage abundance and impaired post-ischemic repair in ICM. The retinoic acid-induced tumor suppressor (47) retinoic acid receptor responder 1 (RARRES1), also known as tazarotene-induced gene 1 (TIG1), was among the most frequently methylated loci (48) in human cancers and was linked to malignant progression (49). Its role in cardiovascular diseases remained unclear. One study identified RARRES1 as part of a urinary proteomic panel for diagnosing right ventricular dysfunction in patients with idiopathic dilated cardiomyopathy (DCM) (50). Notably, that study employed urinary proteomics, whereas we performed a transcriptomics analysis in left ventricular myocardium. For the first time, we demonstrated an association between RARRES1 and ischemic cardiomyopathy.

Our study revealed the CCCTC-binding factor (CTCF) as a candidate transcriptional regulator of ICM. The CTCF protein, which contained 11 evolutionarily conserved zinc finger domains, played critical roles in maintaining chromatin architecture and orchestrating transcriptional domains (51, 52). Prior work linked CTCF dysfunction to various diseases, including cancers (53), and neurodevelopmental disorders (54), and was also associated with aberrant cardiac development. Specifically, the homozygous CTCF-R567W mutation caused significant impairments in the mouse embryonic heart (55). CTCF was reported to be at low levels in blood samples from children with heart failure (56), consistent with our observation of reduced CTCF expression in the ICM. The same research also demonstrated that the overexpression of CTCF weakened tunicamycin-induced ER stress and apoptosis in cardiomyocytes through the S100A1-RYR2 axis (56). Additionally, CTCF mediated Gs*α*-induced NRF2 activation to suppress ferroptosis, thereby alleviating endothelial dysfunction and atherosclerosis (57). Notably, the epigenetic downregulation of RARRES1 was attributed to concurrent alterations in proximal promoter methylation status and CTCF binding deficiency (58).

Due to experimental constraints, we did not initially validate all 329 predicted miRNAs in ICM. Based on our literature search and analysis (see Supplementary Material 3), and to balance success rate with novelty, we selected two miRNAs from the 32 predicted to target RARRES1 for experimental validation: miR-195-5p, which has been previously implicated in heart studies, and miR-5680, which represents a completely unexplored candidate in cardiac research. Notably, miR-195-5p has been reported to promote carotid artery stenosis progression by enhancing vascular smooth muscle cell proliferation and migration (59), and it has also been shown to attenuate the inflammation, apoptosis, oxidative stress, and endoplasmic reticulum stress associated with sepsis-induced myocardial injury (60), suggesting a context-dependent role in cardiovascular pathophysiology. Mechanistically, the isoform miR-195-3p drives cardiac fibrosis and apoptosis via the BDNF/P-ERK1/2 pathway, while the broader miR-195 family participates in complex lncRNA-miRNA-mRNA networks, such as the XIST/miR-195-5p/caspase-1 axis, which regulates inflammatory pyroptosis (61, 62). The stability of miR-195 offers emerging potential in forensic medicine for estimating time-since-death or cause-of-death in cardiac fatalities, highlighting its multifaceted clinical relevance (63). Kuai et al. conducted their analysis based on serum samples from the China National Heart Failure Registry (CN-HF), demonstrating that miR-195-5p levels were significantly decreased in heart failure patients with reduced ejection fraction (64), contradicting our observations in the ICM model. We propose that this inconsistency likely reflected tissue-specific expression heterogeneity between circulating blood and myocardial tissues. Although miR-5680 has no prior cardiac evidence, preliminary studies have implicated it in gastric cancer lymphatic metastasis (65). We demonstrated for the first time that miR-5680 expression was elevated in the myocardial tissues of ischemic cardiomyopathy. The inverse expression patterns of miR-5680, miR-195-5p, and RARRES1 aligned with our computational prediction that both miRNAs potentially target RARRES1.

Although integrated single-cell RNA sequencing data detected no significant differences in MLLT3 and GFOD1 mRNA expression levels between the NC and ICM groups, our animal experiments indicate that GFOD1, MLLT3, COLEC12, and RARRES1, along with miR-195-5p and miR-5680, and the transcription factor CTCF, are significantly associated with ischemic cardiomyopathy. This discrepancy may be attributable to the inherent differences in gene detection sensitivity across the four scRNA-seq validation datasets (see Supplementary Table 1). The convergent identification of these genes across independent studies and distinct biological matrices strengthens, rather than diminishes, their potential as cardiomyopathy-related biomarkers. More importantly, our study is the first to report the downregulation of CTCF and the upregulation of miR-5680 in ischemic cardiomyopathy, providing novel candidate targets for future mechanistic and therapeutic investigations.

Distinct expression of these hub genes across different cell types was observed. GFOD1 was predominantly enriched in endothelial cells, whereas COLEC12 and RARRES1 exhibited fibroblast-epicardial specificity, corroborating prior reports of fibroblast activation in myocardial fibrosis (66, 67). Notably, we found that low hub gene-expressing cell populations were enriched in pathways related to cytoskeletal remodeling (e.g., hypertrophic cardiomyopathy) and inflammatory responses (e.g., AGE-RAGE signaling), which aligned with known mechanisms of ischemic cardiomyopathy. Furthermore, we identified specific transcriptional regulator (CTCF) and miRNAs (miR-195-5p, miR-5680) that may modulate these genes, unveiling a regulatory mechanism previously uncharacterized in the ICM. Our ICM rat model validated the dysregulation of CTCF and candidate miRNAs (miR-195-5p, miR-5680). Therapeutic targeting of these regulators may represent a novel strategy to attenuate pathological myocardial remodeling in ICM.

Our results also raise new questions and hypotheses. For instance, the shared transcription factor CTCF, which regulates multiple hub genes, may play a central role in ICM pathogenesis. This finding aligns with studies implicating CTCF in chromatin organization and cardiac development, yet our work is the first to link CTCF to ICM progression. Future studies could investigate the mechanisms by which CTCF regulates these genes and their downstream pathways. MiRNAs have been shown to play critical roles in cardiac pathology, and their modulation have been explored as a therapeutic strategy in cardiovascular diseases. Similarly, miR-195-5p, previously associated with cardiac hypertrophy, emerges here as a potential post-transcriptional regulator of RARRES1, highlighting its potential role in ICM pathogenesis. The targets and functional roles of miR-195-5p and miR-5680 in the ICM warrant further exploration. Moreover, do these biomarkers exhibit stage-specific expression patterns during ICM progression? These investigations could provide deeper insights into the molecular mechanisms underlying ICM and inform future mechanistic studies.

Despite these promising results, our study has several limitations. First, our pseudobulk analysis used whole-cell aggregation following major cell subpopulation annotation. Although no significant compositional differences were detected among major subpopulations between groups (see Supplementary Material 5), we cannot rule out potential confounding effects from compositional variations within more refined functional subclusters. Future studies with higher-resolution cellular taxonomy are needed to validate the identified signature genes. Additionally, the cell-type-specific localization of these biomarkers was only demonstrated through bubble plots derived from the GSE145154 dataset without experimental validation via multi-marker immunofluorescence, which represents an important direction for our next-phase investigations. Second, the GSE145154 dataset used for cell-type annotation and pseudobulk analysis included only two non-failing controls. To partially mitigate this, we integrated GSE145154 with four additional normal human cardiac scRNA-seq datasets exclusively for validation of signature gene expression. Notably, we did not use these integrated datasets for initial feature gene screening, as integrating multiple datasets could complicate accurate manual annotation. Nevertheless, this imbalance may limit the generalizability of the reference transcriptional landscape, and future studies with larger control cohorts are needed to confirm these findings. Third, the biomarkers revealed in this study were initially derived from bioinformatic analysis of human ICM tissue samples, subsequently validated in a rat model, and their clinical applicability in human requires further investigation. Moreover, this study used a limited number of rats per group (*n* = 4 for echocardiography and ELISA; *n* = 2 for histological and molecular analyses, with 3 technical replicates per animal). The small sample size may limit statistical power. Therefore, the present findings should be considered preliminary and require independent replication in larger animal cohorts. Fourth, the regulatory networks involving TFs and miRNAs were predicted computationally. While our bioinformatic and *in vivo* expression findings provide correlative evidence, they do not establish causal mechanisms. In addition, our miRNA selection for experimental validation was exploratory and literature-driven, without systematic expression-based pre-screening. Future studies are needed to perform cell-type-specific perturbations, such as siRNA-mediated knockdown of COLEC12 in macrophages and CTCF perturbation in cardiac fibroblasts, as well as luciferase reporter assays to validate the predicted miR-195-5p/miR-5680–RARRES1 interactions. These experiments will be critical to determine whether these genes functionally contribute to ICM pathogenesis. Finally, our scRNA-seq analysis focused on left ventricular samples, and the inclusion of atrial or right ventricular tissues may map regional heterogeneity in ICM pathology.

Overall, these findings provide a multi-layered molecular landscape of ischemic cardiomyopathy, laying a groundwork for future mechanistic dissection. Further validation and functional studies are needed to fully understand their clinical potential.

## Conclusion

In conclusion, this study highlights a panel of novel genes and regulatory molecules associated with ischemic cardiomyopathy, offering promising biomarker candidates for further investigation.

## Data Availability

Publicly available datasets were analyzed in this study. This data can be found here: https://www.ncbi.nlm.nih.gov/geo/query/acc.cgi?acc=gse145154 and https://www.ncbi.nlm.nih.gov/geo/query/acc.cgi?acc=gse5406.
