# Supplementary material for: Single-cell RNA sequencing pseudobulk analysis and machine learning identify candidate biomarkers for ischemic cardiomyopathy
Source: Front Cardiovasc Med. 2026 Jul 29;13:1840776. doi: 10.3389/fcvm.2026.1840776 (PMC13461520; doi:10.3389/fcvm.2026.1840776)
Supplement: Supplementary file 1 [file Datasheet1.zip › Supplementary Material 2/RF/Random forest.pdf]

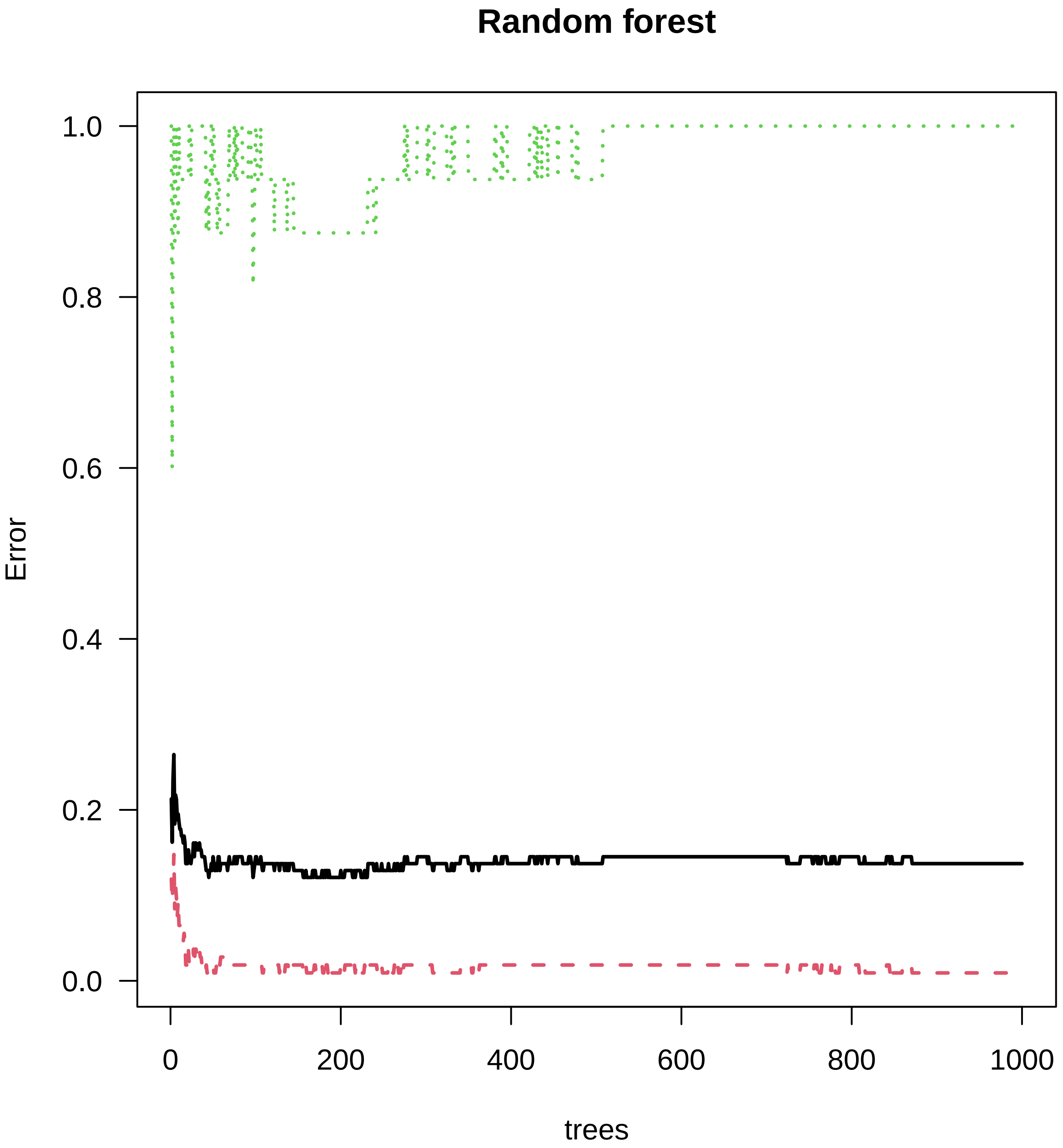
Random forest
Error
1.0
0.8
0.6
0.4
0.2
0.0
0
200
400
600
800
1000
trees
