# Supplementary material for: Single-cell RNA sequencing pseudobulk analysis and machine learning identify candidate biomarkers for ischemic cardiomyopathy
Source: Front Cardiovasc Med. 2026 Jul 29;13:1840776. doi: 10.3389/fcvm.2026.1840776 (PMC13461520; doi:10.3389/fcvm.2026.1840776)
Supplement: Supplementary file 5 [file Datasheet5.zip › Supplementary Material 5/Cell_proportion_no_diff.pdf]

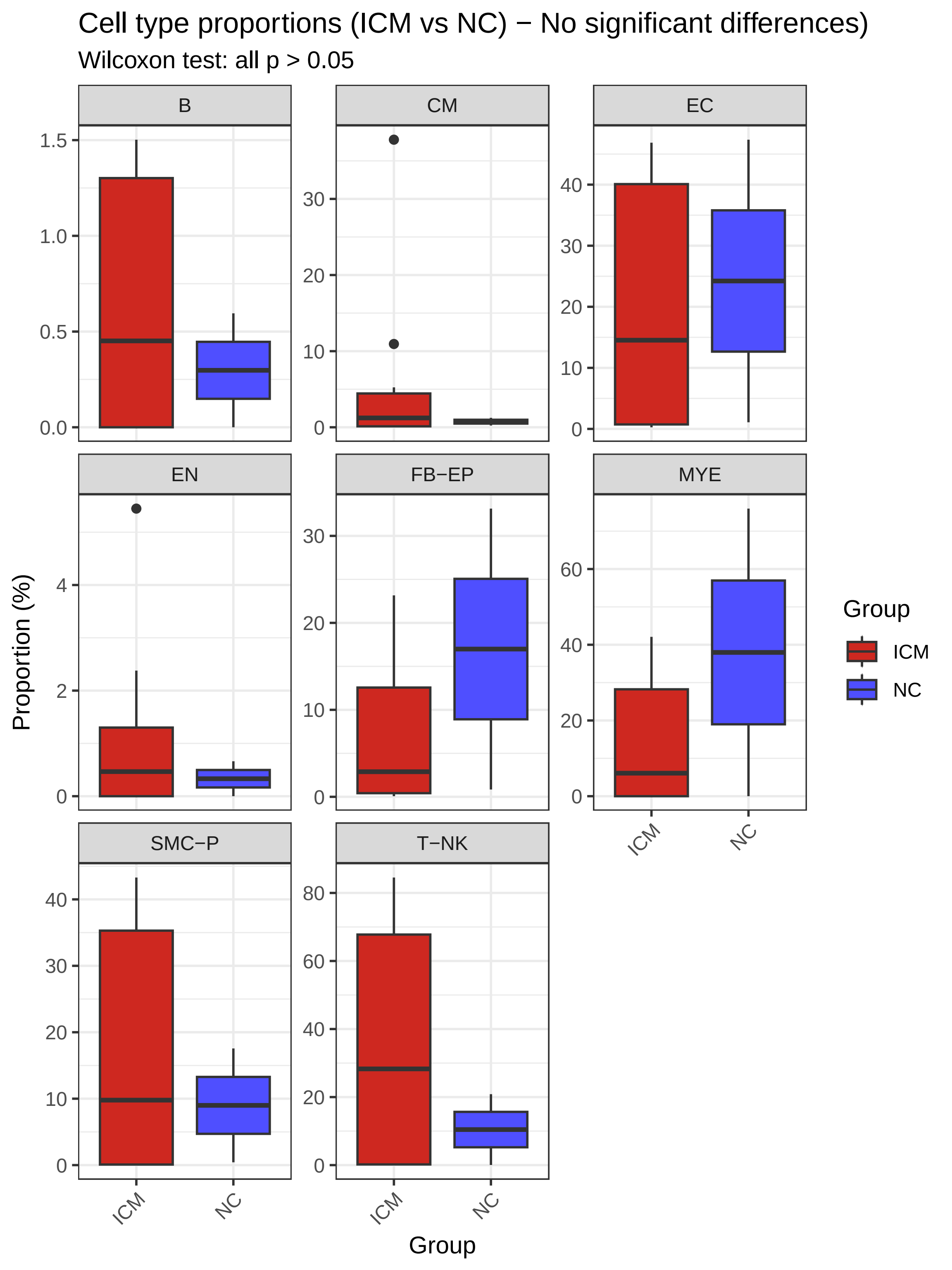

Cell type proportions (ICM vs NC) − No significant differences)
Wilcoxon test: all p > 0.05
B
CM
EC
EN
FB−EP
MYE
SMC−P
T−NK
Proportion (%)
Group
ICM
NC
