# Supplementary material for: Single-cell RNA sequencing pseudobulk analysis and machine learning identify candidate biomarkers for ischemic cardiomyopathy
Source: Front Cardiovasc Med. 2026 Jul 29;13:1840776. doi: 10.3389/fcvm.2026.1840776 (PMC13461520; doi:10.3389/fcvm.2026.1840776)
Supplement: Supplementary file 3 [file Datasheet3.zip › Supplementary Material 3/Literature retrieval and analysis of 33 predicted miRNA targeting to RARRES1.zip!/Search strategy in PubMed.docx]

**Search strategy in PubMed**

**1	miR-195-5p**

("microRNA-195"[MeSH Terms] OR "miR-195-5p"[Title/Abstract] OR "mir-195-5p"[Title/Abstract] OR "hsa-mir-195-5p"[Title/Abstract] OR "miR-195"[Title/Abstract]) AND ("Heart"[MeSH Terms] OR "Cardiac"[Title/Abstract] OR "Myocardial"[Title/Abstract] OR "Heart Diseases"[MeSH Terms] OR "Cardiovascular"[Title/Abstract])

**2	miR-206**

("MicroRNAs"[MeSH Terms] OR "microRNA"[Text Word] OR "miRNA"[Text Word] OR "miR-206"[Text Word] OR "mir206"[Text Word] OR "MIR206"[Text Word]) AND ("Heart"[MeSH Terms] OR "Cardiac"[Text Word] OR "Myocardial"[Text Word] OR "Heart Diseases"[MeSH Terms] OR "Cardiovascular"[Text Word] OR "Ventricular"[Text Word] OR "Atrial"[Text Word])

**3	miR-30a-5p**

("microRNA-30a"[MeSH Terms] OR "miR-30a-5p"[Title/Abstract] OR "hsa-miR-30a-5p"[Title/Abstract] OR "mir30a-5p"[Title/Abstract]) AND ("Heart"[MeSH Terms] OR "Cardiac"[Title/Abstract] OR "Myocardial"[Title/Abstract] OR "Cardiovascular"[Title/Abstract] OR "Heart Diseases"[MeSH Terms])

**4	miR-30b-5p**

("microRNA-30b"[MeSH Terms] OR "miR-30b-5p"[Title/Abstract] OR "hsa-mir-30b-5p"[Title/Abstract] OR "mir-30b-5p"[Title/Abstract]) AND ("Heart"[MeSH Terms] OR "Cardiac"[Title/Abstract] OR "Myocardial"[Title/Abstract] OR "Cardiovascular"[Title/Abstract] OR "Heart Diseases"[MeSH Terms])

**5	miR-30c-5p**

("MicroRNAs"[MeSH Terms] OR "microRNA"[Title/Abstract] OR "miRNA"[Title/Abstract] OR "miR-30c-5p"[Title/Abstract] OR "hsa-mir-30c-5p"[Title/Abstract] OR "MIR30C"[Title/Abstract]) AND ("Heart"[MeSH Terms] OR "Cardiac"[Title/Abstract] OR "Myocardial"[Title/Abstract] OR "Cardiovascular"[Title/Abstract] OR "Heart Diseases"[MeSH Terms] OR "Cardiomyopathy"[MeSH Terms] OR "Heart Failure"[MeSH Terms])

**6	miR-30d-5p**

("MicroRNAs"[MeSH Terms] OR "microRNA"[Title/Abstract] OR "miRNA"[Title/Abstract] OR "miR-30d-5p"[Title/Abstract] OR "hsa-mir-30d-5p"[Title/Abstract] OR "MIR30D"[Title/Abstract]) AND ("Heart"[MeSH Terms] OR "Cardiac"[Title/Abstract] OR "Myocardial"[Title/Abstract] OR "Cardiovascular"[Title/Abstract] OR "Heart Diseases"[MeSH Terms] OR "Cardiomyopathy"[MeSH Terms])

**7	miR-30e-5p**

("microRNAs"[MeSH Terms] OR "microRNA"[Title/Abstract] OR "miRNA"[Title/Abstract] OR "miR-30e-5p"[Title/Abstract] OR "hsa-miR-30e-5p"[Title/Abstract]) AND ("Heart"[MeSH Terms] OR "Cardiac"[Title/Abstract] OR "Myocardial"[Title/Abstract] OR "Cardiovascular"[Title/Abstract] OR "Heart Diseases"[MeSH Terms])

**8	miR-3146**

("microRNA-3146"[MeSH Terms] OR "miR-3146"[Title/Abstract] OR "mir3146"[Title/Abstract] OR "hsa-mir-3146"[Title/Abstract]) AND ("Heart"[MeSH Terms] OR "Cardiac"[Title/Abstract] OR "Myocardial"[Title/Abstract] OR "Cardiovascular"[Title/Abstract] OR "Heart Diseases"[MeSH Terms])

**9	miR-32-3p**

("MicroRNAs"[MeSH Terms] OR "microRNA"[Title/Abstract] OR "miRNA"[Title/Abstract] OR "miR-32-3p"[Title/Abstract] OR "hsa-miR-32-3p"[Title/Abstract]) AND ("Heart"[MeSH Terms] OR "Cardiac"[Title/Abstract] OR "Myocardial"[Title/Abstract] OR "Cardiovascular"[Title/Abstract] OR "Heart Diseases"[MeSH Terms])

**10	miR-3942-5p**

("MicroRNAs"[MeSH Terms] OR "microRNA"[Title/Abstract] OR "miRNA"[Title/Abstract] OR "miR-3942-5p"[Title/Abstract] OR "hsa-mir-3942-5p"[Title/Abstract] OR "MIR3942-5p"[Title/Abstract]) AND ("Heart"[MeSH Terms] OR "Cardiac"[Title/Abstract] OR "Myocardial"[Title/Abstract] OR "Cardiovascular"[Title/Abstract] OR "Heart Diseases"[MeSH Terms])

**11	miR-4282**

("MicroRNAs"[MeSH Terms] OR "microRNA"[Title/Abstract] OR "miRNA"[Title/Abstract] OR "miR-4282"[Title/Abstract] OR "hsa-mir-4282"[Title/Abstract]) AND ("Heart"[MeSH Terms] OR "Cardiac"[Title/Abstract] OR "Myocardial"[Title/Abstract] OR "Heart Diseases"[MeSH Terms] OR "Cardiovascular"[Title/Abstract])

**12	miR-4682**

("MicroRNAs"[MeSH Terms] OR "microRNA"[Title/Abstract] OR "miRNA"[Title/Abstract] OR "miR-4282"[Title/Abstract] OR "hsa-mir-4282"[Title/Abstract]) AND ("Heart"[MeSH Terms] OR "Cardiac"[Title/Abstract] OR "Myocardial"[Title/Abstract] OR "Heart Diseases"[MeSH Terms] OR "Cardiovascular"[Title/Abstract])

13	**miR-4729**

("microRNA-4729"[MeSH Terms] OR "miR-4729"[Title/Abstract] OR "mir4729"[Title/Abstract] OR "hsa-mir-4729"[Title/Abstract]) AND ("Heart"[MeSH Terms] OR "Cardiac"[Title/Abstract] OR "Cardiovascular"[Title/Abstract] OR "Myocardial"[Title/Abstract] OR "Heart Diseases"[MeSH Terms] OR "Cardiomyopathy"[MeSH Terms])

14	**miR-4786-5p**

("MicroRNAs"[MeSH Terms] OR "microRNA"[Title/Abstract] OR "miRNA"[Title/Abstract] OR "mir-4786-5p"[Title/Abstract] OR "hsa-mir-4786-5p"[Title/Abstract] OR "miR-4786-5p"[Title/Abstract]) AND ("Heart"[MeSH Terms] OR "cardiac"[Title/Abstract] OR "heart"[Title/Abstract] OR "myocardial"[Title/Abstract] OR "cardiovascular"[Title/Abstract] OR "ventricle"[Title/Abstract] OR "atrium"[Title/Abstract] OR "cardiomyocyte"[Title/Abstract])

15	**miR-539-5p**

("microRNA-539"[MeSH Terms] OR "miR-539-5p"[Title/Abstract] OR "mir-539-5p"[Title/Abstract] OR "hsa-mir-539-5p"[Title/Abstract]) AND ("Heart"[MeSH Terms] OR "cardiac"[Title/Abstract] OR "heart disease"[Title/Abstract] OR "myocardial"[Title/Abstract] OR "cardiovascular"[Title/Abstract])

16	**miR-548a-5p**

("microRNA-548a-5p"[MeSH Terms] OR "miR-548a-5p"[Title/Abstract] OR "mir548a5p"[Title/Abstract] OR "hsa-mir-548a-5p"[Title/Abstract]) AND ("Heart"[MeSH Terms] OR "Cardiac"[Title/Abstract] OR "Myocardial"[Title/Abstract] OR "Cardiovascular"[Title/Abstract] OR "Heart Diseases"[MeSH Terms])

17	**miR-548b-5p**

("MicroRNAs"[MeSH Terms] OR "microRNA"[Title/Abstract] OR "miRNA"[Title/Abstract] OR "micro ribonucleic acid"[Title/Abstract]) AND ("miR-548b-5p"[Title/Abstract] OR "hsa-mir-548b-5p"[Title/Abstract] OR "mir548b-5p"[Title/Abstract] OR "MIR548B-5p"[Title/Abstract]) AND ("Heart"[MeSH Terms] OR "Cardiac"[Title/Abstract] OR "Myocardial"[Title/Abstract] OR "Cardiovascular"[Title/Abstract] OR "Heart Diseases"[MeSH Terms] OR "Cardiomyopathy"[MeSH Terms] OR "Arrhythmia"[MeSH Terms] OR "Heart Failure"[MeSH Terms])

18	**miR-548c-5p**

("microRNA-548c-5p"[MeSH Terms] OR "miR-548c-5p"[Title/Abstract] OR "mir-548c-5p"[Title/Abstract] OR "hsa-mir-548c-5p"[Title/Abstract]) AND ("Heart"[MeSH Terms] OR "Cardiac"[Title/Abstract] OR "Myocardial"[Title/Abstract] OR "Cardiovascular"[Title/Abstract] OR "Heart Diseases"[MeSH Terms] OR "Cardiomyopathy"[MeSH Terms])

19	**miR-548d-5p**

("MicroRNAs"[MeSH Terms] OR "microRNA"[Title/Abstract] OR "miRNA"[Title/Abstract] OR "miR-548d-5p"[Title/Abstract] OR "hsa-mir-548d-5p"[Title/Abstract]) AND ("Heart"[MeSH Terms] OR "Cardiac"[Title/Abstract] OR "Heart"[Title/Abstract] OR "Myocardial"[Title/Abstract] OR "Cardiovascular"[Title/Abstract] OR "Left Ventricle"[MeSH Terms] OR "Right Ventricle"[MeSH Terms] OR "Atrium"[MeSH Terms] OR "Ventricle"[MeSH Terms])

20	**miR-548h-5p**

("MicroRNAs"[MeSH Terms] OR "microRNA"[Title/Abstract] OR "miRNA"[Title/Abstract] OR "miR-548h-5p"[Title/Abstract] OR "hsa-miR-548h-5p"[Title/Abstract]) AND ("Heart"[MeSH Terms] OR "Cardiac"[Title/Abstract] OR "Heart"[Title/Abstract] OR "Myocardial"[Title/Abstract] OR "Cardiovascular"[Title/Abstract])

21	**miR-548i**

("MicroRNAs"[MeSH Terms] OR "microRNA"[Title/Abstract] OR "miRNA"[Title/Abstract] OR "miR-548i"[Title/Abstract] OR "mir548i"[Title/Abstract] OR "hsa-mir-548i"[Title/Abstract]) AND ("Heart"[MeSH Terms] OR "Cardiac"[Title/Abstract] OR "Heart"[Title/Abstract] OR "Myocardial"[Title/Abstract] OR "Cardiovascular"[Title/Abstract] OR "Ventricular"[Title/Abstract] OR "Atrial"[Title/Abstract])

22	**miR-548j-5p**

("microRNA-548j-5p"[MeSH Terms] OR "miR-548j-5p"[Title/Abstract] OR "mir-548j-5p"[Title/Abstract] OR "hsa-mir-548j-5p"[Title/Abstract]) AND ("Heart"[MeSH Terms] OR "Cardiac"[Title/Abstract] OR "Myocardial"[Title/Abstract] OR "Cardiovascular"[Title/Abstract] OR "Heart Diseases"[MeSH Terms])

23	**miR-548k**

("microRNA-548k"[MeSH Terms] OR "miR-548k"[Title/Abstract] OR "mir548k"[Title/Abstract] OR "hsa-mir-548k"[Title/Abstract] OR "hsa-miR-548k"[Title/Abstract]) AND ("Heart"[MeSH Terms] OR "Cardiac"[Title/Abstract] OR "Cardiovascular"[Title/Abstract] OR "Myocardial"[Title/Abstract] OR "Heart Diseases"[MeSH Terms] OR "Cardiomyopathy"[MeSH Terms] OR "Heart Failure"[MeSH Terms])

24	**miR-548q**

("MicroRNAs"[MeSH Terms] OR "microRNA"[Title/Abstract] OR "miRNA"[Title/Abstract] OR "miR-548q"[Title/Abstract] OR "mir-548q"[Title/Abstract]) AND ("Heart"[MeSH Terms] OR "Cardiac"[Title/Abstract] OR "Myocardial"[Title/Abstract] OR "Heart Diseases"[MeSH Terms] OR "Cardiovascular"[Title/Abstract])

25	**miR-548w**

("microRNA-548w"[MeSH Terms] OR "miR-548w"[Title/Abstract] OR "mir548w"[Title/Abstract] OR "hsa-mir-548w"[Title/Abstract] OR "hsa-miR-548w"[Title/Abstract]) AND ("Heart"[MeSH Terms] OR "Cardiac"[Title/Abstract] OR "Cardiovascular"[Title/Abstract] OR "Myocardial"[Title/Abstract] OR "Heart Diseases"[MeSH Terms] OR "Cardiovascular Diseases"[MeSH Terms])

26	**miR-548y**

("microRNAs"[MeSH Terms] OR "microRNA"[Title/Abstract] OR "miRNA"[Title/Abstract] OR "miR-548y"[Title/Abstract] OR "mir-548y"[Title/Abstract] OR "hsa-mir-548y"[Title/Abstract]) AND ("Heart"[MeSH Terms] OR "Cardiac"[Title/Abstract] OR "Cardiovascular"[Title/Abstract] OR "Myocardial"[Title/Abstract] OR "Heart Diseases"[MeSH Terms] OR "Cardiomyopathy"[MeSH Terms] OR "Arrhythmia"[MeSH Terms] OR "Heart Failure"[MeSH Terms])

27	**miR-553**

("microRNA-553"[MeSH Terms] OR "miR-553"[Title/Abstract] OR "hsa-mir-553"[Title/Abstract] OR "mir553"[Title/Abstract]) AND ("Heart"[MeSH Terms] OR "Cardiac"[Title/Abstract] OR "Myocardial"[Title/Abstract] OR "Cardiovascular"[Title/Abstract] OR "Heart Diseases"[MeSH Terms])

28	**miR-556-3p**

("microRNAs"[MeSH Terms] OR "microRNA"[Title/Abstract] OR "miRNA"[Title/Abstract] OR "miR-556-3p"[Title/Abstract] OR "hsa-miR-556-3p"[Title/Abstract]) AND ("Heart"[MeSH Terms] OR "Cardiac"[Title/Abstract] OR "Myocardial"[Title/Abstract] OR "Cardiovascular"[Title/Abstract] OR "Heart Diseases"[MeSH Terms] OR "Cardiomyopathy"[MeSH Terms])

**29	miR-5680**

("microRNA-5680"[MeSH Terms] OR "miR-5680"[Title/Abstract] OR "mir5680"[Title/Abstract] OR "hsa-mir-5680"[Title/Abstract]) AND ("Heart"[MeSH Terms] OR "Cardiac"[Title/Abstract] OR "Cardiovascular"[Title/Abstract] OR "Myocardial"[Title/Abstract] OR "Heart Diseases"[MeSH Terms] OR "Cardiomyopathy"[MeSH Terms] OR "Arrhythmia"[MeSH Terms] OR "Heart Failure"[MeSH Terms])

30	**miR-582-5p**

("microRNA-582"[MeSH Terms] OR "miR-582-5p"[Title/Abstract] OR "hsa-mir-582"[Title/Abstract] OR "mir-582-5p"[Title/Abstract]) AND ("Heart"[MeSH Terms] OR "Cardiac"[Title/Abstract] OR "Myocardial"[Title/Abstract] OR "Cardiovascular"[Title/Abstract] OR "Heart Diseases"[MeSH Terms] OR "Cardiomyopathy"[MeSH Terms])

31	**miR-6500-3p**

("microRNA-6500-3p"[MeSH Terms] OR "mir-6500-3p"[Title/Abstract] OR "hsa-mir-6500-3p"[Title/Abstract] OR "miR-6500-3p"[Title/Abstract]) AND ("Heart"[MeSH Terms] OR "Cardiac"[Title/Abstract] OR "Myocardial"[Title/Abstract] OR "Cardiovascular"[Title/Abstract] OR "Heart Diseases"[MeSH Terms])

32	**miR-674**

("MicroRNAs"[MeSH Terms] OR "microRNA"[Text Word] OR "miRNA"[Text Word] OR "micro ribonucleic acid"[Text Word]) AND ("miR-674"[Text Word] OR "mir-674"[Text Word] OR "hsa-mir-674"[Text Word] OR "has-miR-674"[Text Word] OR "MIR674"[Text Word]) AND ("Heart"[MeSH Terms] OR "Cardiac"[Text Word] OR "Cardiovascular"[Text Word] OR "Myocardial"[Text Word] OR "Heart Diseases"[MeSH Terms] OR "Cardiomyopathy"[MeSH Terms] OR "Heart Failure"[MeSH Terms])

33	**miR-9983-3p**

("MicroRNAs"[MeSH Terms] OR "microRNA"[Title/Abstract] OR "miRNA"[Title/Abstract] OR "miR-9983-3p"[Title/Abstract] OR "hsa-miR-9983-3p"[Title/Abstract] OR "mir-9983-3p"[Title/Abstract]) AND ("Heart"[MeSH Terms] OR "Cardiac"[Title/Abstract] OR "Heart"[Title/Abstract] OR "Myocardial"[Title/Abstract] OR "Cardiovascular"[Title/Abstract] OR "Left Ventricle"[MeSH Terms] OR "Right Ventricle"[MeSH Terms] OR "Atrium"[MeSH Terms] OR "Cardiomyocyte"[Title/Abstract])
